# Protocol for Tri-culture of hiPSC-Derived Neurons, Astrocytes, and Microglia

**DOI:** 10.1016/j.xpro.2020.100190

**Published:** 2020-12-01

**Authors:** Sean K. Ryan, Kelly L. Jordan-Sciutto, Stewart A. Anderson

**Affiliations:** 1 Department of Pathology, Perelman School of Medicine, University of Pennsylvania, Philadelphia, PA 19104, USA; 2 Department of Psychiatry, The Children's Hospital of Philadelphia, Philadelphia, PA 19104, USA; 3 Department of Psychiatry, Perelman School of Medicine, University of Pennsylvania, Philadelphia, PA 19104, USA; 4 Department of Basic and Translational Sciences, School of Dental Medicine, University of Pennsylvania, Philadelphia, PA 19104, USA

**Keywords:** Cell Culture, Neuroscience, Stem Cells, Cell Differentiation

## Abstract

This protocol establishes a tri-culture of hiPSC-derived neurons, astrocytes, and microglia for the study of cellular interactions during homeostasis, injury, and disease. This system allows for mechanistic studies that can identify the roles of individual cell types in disease and injury response in a physiologically relevant, all-human system. This protocol utilizes and modifies prior differentiations. Limitations include the prolonged maturation of human astrocytes and neurons and scalability.

For complete details on the use and execution of this protocol, please refer to Ryan et al. (2020).

## Highlights

• 30-day tri-culture assembly once iAstrocytes and CMPs are produced
• Can integrate disease iPSC lines for one to all three cell types
• Reduced and tractable system allowing for mechanistic studies
• Allows for better control of cell-type ratios than organoids

## Graphical Abstract

**Figure:**
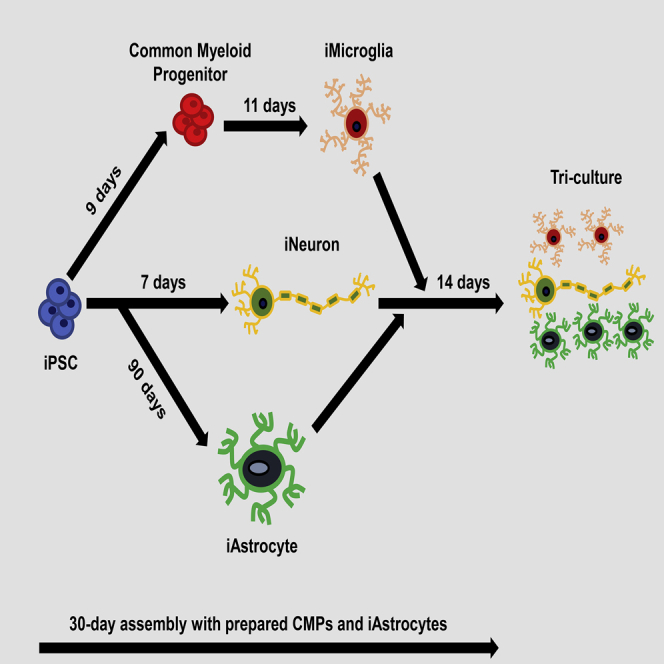

## Before You Begin

In preparation for the astrocyte and neuron differentiations, hiPSCs must be transduced with the TetOn-NGN2 and rTTA viruses to allow for conditional activation of the transcription factor NGN2. This section of the protocol was based on previously published work (Zhang et al., 2013). Although, other neuronal subclasses could be used in this protocol.

### Transduce and Expand hiPSC Lines with NGN2 and rTTA Lentiviruses

**Timing: 5–7 weeks**

1. Thaw Matrigel on ice.

***Note:*** Thawing Matrigel too quickly or letting get too warm will cause it to polymerize. Make sure to keep everything cold until the plates are coated.

2. Add thawed Matrigel to 4°C DMEM.
3. Coat 6-well plates with cold 1:50 (Matrigel: DMEM/F12) and then incubate in 37°C 5% CO_2_ incubator for at least 30 min, but no more than 8 h. This incubation time should be followed for all Matrigel coatings throughout this protocol.
4. Seed hiPSCs as single cells onto 1:50 (Matrigel: DMEM/F12) Matrigel-coated 6-well plates at 5 × 10^4^ cells/well (5.2 × 10^3^ cells/cm^2^) in Stem MACS iPS-Brew XF media with 10 μM Y27632 (1:1,000 of 10 mM stock in DMSO).
5. Allow cells to adhere for 24 h in a 37°C 5% CO_2_ incubator.
6. Prepare a beaker of 10% bleach in the culture hood.
7. Thaw aliquots of 1 μg/μL polybrene and at least 8 μL of each rTTA and TetO-NGN2 lentivirus.

***Note:*** virus stocks were donated by Marius Wernig (Stanford University) and amplified by the Penn Vector Core. However, versions of these viruses can be ordered from Addgene.

***Note:*** NGN2 titer was 8.81 × 10^10^ IU/mL and rTTA titer was 3.54 × 10^10^ IU/mL.

***Note:*** There was variability in infection rate from line to line. This will need to be tested empirically as described below.

8. Replace stem cell media with standard Stem MACS iPS-Brew.
9. In each of the 6 wells, add 4 μL of the 1 μg/μL stock of polybrene to the 2 mL of Stem MACS media.
10. In 5 of the 6 wells, leaving one as a polybrene-only control add the following amounts of EACH virus: 0.25, 0.5, 1, 2, and 4 μL.
11. Dispose of all excess virus and all pipettes that have touched the media and viruses in 10% bleach or inactivate lentiviral particles according to local regulations.
12. Wait 6 h with cells in 37°C 5% CO_2_ incubator, then replace media with fresh Stem MACS iPS-Brew XF media.
  a. All media and pipette tips must be disposed of in 10% bleach.
13. Evaluate cell survival and perform full media exchanges on the cells after 24 and 48 h. The well exposed to the highest concentration of virus that survived the infection can be taken and expanded.
14. Expand and freeze down at least 50 vials per line (2 million cells per vial) as a master stock.
  a. Once cells reach ∼80% confluency remove Stem MACS and wash 1× with 20°C PBS.
  b. Aspirate PBS from hiPSCs and apply warm Accutase at half the normal amount of media in the plate (i.e., 1 mL per well of a 6 well plate).
  c. Incubate in 37°C 5% CO_2_ incubator until cells begin lifting from plate (roughly 5 min).
  d. Apply a volume of 37°C DMEM:F-12 equal to that of the Accutase to inactivate the Accutase.
  e. Lift cells from the plate by pipetting up and down slowly with a serological pipette.
  f. Place cells in an appropriately sized conical tube and spin for 5 min at 162 × *g* 20°C.
  g. Aspirate supernatant.
  h. Resuspend in Stem MACS by slowly adding media to the tube and then gently pipetting up and down with a 10 mL pipette until the pellet has uniformly gone into suspension.
  i. Re-plate cells at 1:12.
  j. Expand to at least 50 wells of 6 well plates.
  k. Freeze down at least 50 wells by lifting and spinning hiPSCs as just described.
  l. Count cells by hemocytometer or vicell or equivalent method.
  m. Resuspend in Freezing media (10% DMSO in Stem MACS) at 1 mL per 2 × 10^6^ cells.
  n. Freeze down 1 mL per vial and store in a Mr. Frosty or equivalent storage container at −80°C freezer for at least 24 h before moving to LN2 for long term storage.

***Note:*** Freezing down 50+ vials will allow sufficient vials to perform a study all from the same batch of hiPSCs. This will prevent issues arising from random mutations that may be introduced during passage as well as contamination that may occur.

**Figure 1:**
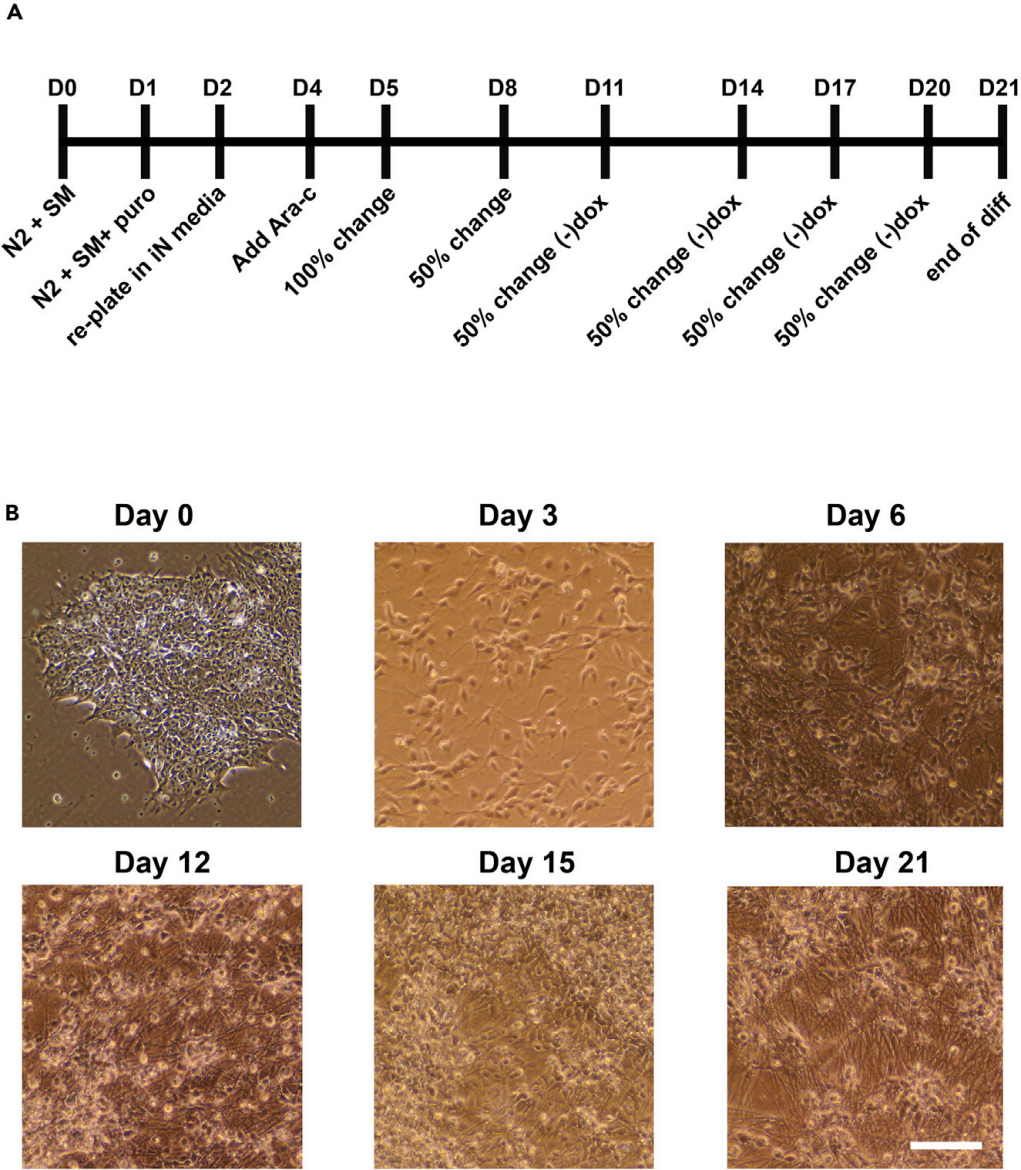
Representative Image of Day 21 iNeurons (A) Timeline of iNeuron differentiation. (B) Representative images of iNeurons in mono-culture through the 21 day differentiation. Scale bar, 50 μm.
21. (Day 1) After 24 h, exchange N2 media + small molecules (BDNF 10 ng/mL, NT3 10 ng/mL, laminin 200 ng/mL, doxycycline 2 μg/mL) with 15 mL fresh N2 media + small molecules supplemented with 5 μg/mL puromycin (stock concentration: 10 mg/mL in H_2_O).

***Note:*** The virus that was used was on a puromycin resistance background. We selected for transduced cells with puromycin. Pending on the selection tool for the various forms of the virus from commercial vendors, puromycin may not be necessary.

15. Test transduction efficiency by starting a small-scale hiPSC-derived neuron (iNeuron) Differentiation.
16. Thaw a vial of transduced hiPSCs and plate onto three wells of a 1:50 (Matrigel: DMEM:F-12) Matrigel-coated 6-well plate (7 × 10^4^ cells/cm^2^) in Stem MACS iPS-Brew XF media with 10 μM Y27632.
17. Replace media the following day without Y27632.
  a. Continue to feed daily until cells reach ∼80% confluency (1 to 2 days).

18. Once ∼80% confluent, split (as described below) cells to 1.2 × 10^4^ cells/cm^2^ on a 10 cm dish (roughly 1 confluent well of a 6 well can be split to 3 × 10 cm plates).
  ***Note:*** A single passage post thaw is sufficient.
  a. Remove Stem MACS and wash 1× with 20°C PBS.
  b. Aspirate PBS from hiPSCs and apply warm Accutase (pre-warmed in 37°C water bath for 15 min) at half the normal amount of media in the plate (i.e., 1 mL per well of a 6 well plate).
  c. Incubate in 37°C 5% CO_2_ incubator until cells begin lifting from plate (roughly 5 min).
  d. Apply a volume of 37°C DMEM:F-12 equal to that of the Accutase used.
  e. Wash cells from bottom of plate by pipetting up and down slowly three times with a 5 mL serological pipette.
  f. Place cells in an appropriately sized conical tube and spin for 5 min at 162 × *g* 20°C.
  g. Resuspend in Stem MACS by slowly adding media to the tube and then gently pipetting up and down with a 10 mL pipette until the pellet has uniformly gone into suspension. Plate at 1.2 × 10^4^ cells/cm^2^.

19. Full exchange 10 mL of Stem MACS media daily.
20. (Day 0) Once cells reach ∼60% confluency (2–4 days depending on the line), exchange Stem MACS iPS-Brew with 15 mL N2 media + small molecules (BDNF 10 ng/mL, NT3 10 ng/mL, laminin 200 ng/mL, doxycycline 2 μg/mL) (Figures 1A and 1B)

22. (Day 2) After 24 h, lift the cells with Accutase and count.
  ***Note:*** Line-to-line variability can account for great differences in yield. However, a 10 cm plate should yield between 6 million and 12 million NPC-like cells.
  a. Re-plate cells to a 1:20 Matrigel: DMEM/F12 coated plate at 1 × 10^5^ cells/cm^2^.
    i. ***Note:*** seeding density will need to be empirically determined for each line / transduction. However, most cell lines operate well at this density.
    ii. ***Note:*** a 1:20 Matrigel coating increased adherence of neurons through differentiation.

23. (Day 4) Exchange 50% of iN media (neurobasal-A + 1× B27 supplement with vitamin A, 1× glutamax, 5 mM glucose, 10 mM sodium pyruvate, 10 ng/mL BDNF, 10 ng/mL NTF, 2 μg/mL doxycycline) with iN media for maintenance supplemented with 4 μM Ara-c (stock concentration: 10 mM in H_2_O) for a final concentration of 2 μM Ara-c.

***Note:*** We have found that a small percentage of cells that are not transduced survive puromycin selection. If unchecked, they can take over the plate as they are mitotic. In order to remove these cells, Ara-c is added, which will kill dividing cells.

24. (Day 5) Perform a full media exchange to remove the Ara-c.
25. (Day 8) Exchange 50% of iNrn media for maintenance.

26. (Days 11, 14, 17, 20) Exchange 50% of iNrn media without Doxycycline.
  ***Note:*** Doxycycline is discontinued after the day 8 media exchange.
  a. Differentiation ends on day 21, or it can be extended (Figures 1A and 1B).
  b. Immunostain cells for MAP2, synaptophysin, and PSD95 as a measure of quality control.
    i. Greater than 90% of the cells should be positive for these markers.
    ii. ***Note:*** At D21, without astrocyte support, there is very little electrical activity.

## Key Resources Table

**Table:** 

REAGENT or RESOURCE	SOURCE	IDENTIFIER
**Antibodies**		

CX3CR1 (rabbit) (1:500)	Abcam	ab8021
Glutamine synthetase (mouse) (1:500)	Millipore	MAB302
IBA1 (rabbit) (1:500)	WAKO	019-19741
MAP2 (chicken) (1:500)	Abcam	ab5392
P2RY12 (rabbit) (1:100)	Alomone Labs	APR-020
PSD95 clone K28/43 (mouse) (1:500)	NeuroMab	75-028
SOX9 (rabbit) (1:250)	Abcam	ab185230
Synaptophysin clone SY38 (mouse) (1:250)	Millipore	MAB5258
Thrombospondin-1 (rabbit) (1:250)	Abcam	ab85762
TMEM119 (rabbit)	Abcam	ab185333

**Bacterial and Virus Strains**		

VSVG.HIV.SIN.cPPT.CMV.mNgn2.WPR	Wernig lab, Stanford University	received directly from Wernig lab (Addgene #52047)
VSVG.HIV.SIN.cPPT.CMV.rTTA.WPRE	Wernig lab, Stanford University	received directly from Wernig lab (Addgene alternative #19780)

**Chemicals, Peptides, and Recombinant Proteins**		

Ascorbic acid	Sigma-Aldrich	A92902
Astrocyte media	ScienCell	1801
B-27 supplement	Thermo Fisher Scientific	17504044
B-27 supplement w/o retinoic acid	Invitrogen	12587-010
β-Mercaptoethanol	Life Technologies	21985-023
BMP4	Peprotech	120-05
Bovine serum albumin (BSA)	Sigma	A1470
Characterized fetal bovine serum	Hyclone	SH30071.03HI
CHiR 99021	Tocris	4423
CSF-1 recombinant human protein	Thermo Fisher Scientific	PHC9504
Cytosine β-D-arabinofuranoside (Ara-c)	Sigma-Aldrich	C1768
D-(+)-Glucose	Sigma-Aldrich	G8270
DAPI (4′,6-diamidino-2-phenylindole, dilactate)	Thermo Fisher Scientific	Cat# D3571; RRID:AB_2307445
Dispase (1 U/mL)	STEMCELL Technologies	07923
DMEM/F12	Gibco	11320-033
Doxycycline hydrochloride	Sigma-Aldrich	D3072
FGF-2	R&D Systems	233-FB-025
Flt3L	Peprotech	300-19
Glutamax	Life Technologies	35050-061
Hams F-12 medium	Corning	10-080-CV
HyClone RPMI 1640 media	GE Healthcare Life Sciences	SH30027.01
IMDM	Thermo Fisher Scientific	12440053
Laminin from Engelbreth-Holm-Swarm murine sarcoma basement membrane	Sigma-Aldrich	L2020
Matrigel GFR	Corning	354230
Monothioglycerol (MTG)	Sigma-Aldrich	M6145
N2 supplement	Invitrogen	17502-048
N2 supplement B	STEMCELL Technologies	07156
Neurobasal-A	Invitrogen	A24775-01
Penicillin/streptomycin	Thermo Fisher Scientific	15140-148
Polybrene	Sigma-Aldrich	TR-1003
Primocin	Invivogen	Ant-pm-2
Puromycin dihydrochloride	Sigma-Aldrich	P9620
Recombinant human/murine/rat BDNF	Peprotech	450-02
Recombinant human EGF	R&D Systems	236-EG-200
Recombinant human IL-34	R&D Systems	5265-IL-010
Recombinant human NT-3	Peprotech	450-03
Recombinant human TGF-β1	Peprotech	100-21
RPMI 1640	Corning	10-041-CV
RPMI 1640 media	GE Healthcare Life Sciences	SH30027.01
Sodium pyruvate	Sigma-Aldrich	P5280
SCF	Peprotech	300-07
Stem MACS iPS-Brew XF media, human	Miltenyi Biotec	130-104-368
StemPro-34 SFM (1×)	Invitrogen	10639011
StemPro Accutase	Thermo Fisher Scientific	A11105-01
VEGF165	Peprotech	100-20
Y27632	Stemgent	04-2012

**Other**		

CellBIND 24 well clear multiple well plates, flat bottom, with lid, sterile	Corning	3337
Falcon 6 well flat-bottom plates with lids	VWR	62406-161
Falcon 24 well flat-bottom plates with lids	VWR	21100-004
Falcon 100 mm TC-treated cell culture dish	Corning	353003
Nunc Lab-Tek II chamber slide system, sterile, Thermo Scientific (8 well chamber slide)	VWR	62407-296

## Materials and Equipment

**Table:** 

Reagent	Stock Concentration	Final Concentration	Volume
**Base N2 medium (store at 4°C)**			

DMEM:F-12	n/a	n/a	490.5 mL
Glucose	n/a	0.75 g/500 mL	n/a
β-Mercaptoethanol	55 mM	55 μM	500 μL
Primocin	n/a	1:500	1 mL
N2 supplement B (add after filtration step with .22um CA filter)	n/a	1:100	5 mL
Total			50 0mL

**N2 Medium + Small molecules (store at 4°C)**			

Base N2 medium	n/a	n/a	100 mL
BDNF	100 μg/mL	10 ng/mL	10 μL
NT3	100 μg/mL	10 ng/mL	10 μL
Laminin	1 mg/mL	200 ng/mL	20 μL
Doxycycline	100 mg/mL	2 μg/mL	2 μL
Total			100 mL

**Sodium/pyruvate mixture for iN media (store at 4°C)**			

Neurobasal-A	n/a	n/a	20 mL
Glucose	n/a	500 mM	1.8 g
Sodium pyruvate	n/a	1 M	2.21 g
Total	n/a	n/a	20 mL

**iN media for maintenance (store at 4°C)**			

Neurobasal-A	n/a	n/a	97 mL
B27 supplement with vitamin A	50×	1×	2 mL
Glutamax	100×	1×	1 mL
Glucose/sodium pyruvate mixture (100×)	100×	5 mM glucose/10 mM sodium pyruvate	1 mL
BDNF	100 μg/mL	10 ng/mL	10 μL
NT3	100 μg/mL	10 ng/mL	10 μL
Doxycycline	100 mg/mL	2 μg/mL	2 μL
Total	n/a	n/a	100 mL

**High FGF Astrocyte media for first stage maintenance (store at 4°C)**			

N2 base media	n/a	n/a	88mL
FBS	n/a	10%	10mL
B27 with vitamin A	50×	1×	2mL
FGF-2	20 μg/mL	20 ng/mL	100 μL
EGF	20 μg/mL	20 ng/mL	100 μL
Total	n/a	n/a	100 mL

**Low FGF Astrocyte media for second stage maintenance (store at 4°C)**			

N2 base media	n/a	n/a	88 mL
FBS	n/a	10%	10 mL
B27 with vitamin A	50×	1×	2 mL
FGF-2	20 μg/mL	5 ng/mL	25 μL
Total	n/a	n/a	100 mL

**Base RPMI media for CMP differentiation (store at 4°C)**			

Corning 500 mL RPMI 1640	n/a	n/a	97.6 mL
Glutamine	200 mM	2 mM	1 mL
Penicillin/streptomycin	n/a	1:1,000	100 μL
MTG	n/a	400 μM	300 μL
Ascorbic acid	5 mg/mL	50 μg/mL	1 mL
Total	n/a	n/a	100 mL

**Base SP34 media for CMP differentiation (store at 4°C)**			

StemPro-34 SFM (1×)	n/a	n/a	97.6 mL
Glutamine	200 mM	2 mM	1 mL
Penicillin/streptomycin	n/a	1:1,000	100 μL
MTG	n/a	400 μM	300 μL
Ascorbic acid	5 mg/mL	50 μg/mL	1 mL
Total	n/a	n/a	100 mL

**Base SFD media for CMP differentiation (store at 4°C)**			

IMDM	n/a	75%	75 mL
HAMS F-12	n/a	25%	25 mL
N2-supplement	n/a	1:200	500 μL
B27 supplement w/0 retinoic acid	n/a	1:100	1 mL
10% BSA in PBS	n/a	1:200	500 μL
Glutamine	200 mM	2 mM	1 mL
Penicillin/streptomycin	n/a	1:1,000	100 μL
MTG	n/a	400 μM	300 μL
Ascorbic acid	5 mg/mL	50 μg/mL	1 mL
Total	n/a	n/a	104.4 mL

**iMg media for differentiation and maintenance (store at 4°C)**			

HyClone RPMI 1640 media	n/a	n/a	45 mL
Characterized Fetal Bovine Serum, US Origin	n/a	10%	5 mL
Recombinant Human IL-34 Protein	100 μg/mL	100 ng/mL	50 μL
M-CSF Recombinant Human Protein	25 μg/mL	25 ng/mL	50 μL
Recombinant Human TGF-β1	50 μg/mL	50 ng/mL	50 μL
Pen/Strep	n/a	1:1,000	50 μL
Total	n/a	n/a	50 mL

## Step-By-Step Method Details

### Differentiate hiPSCs to Astrocytes

**Timing: 90+ days**

This step will produce a stock of fully differentiated astrocytes that are ready to be added to the tri-culture.

**Figure 2:**
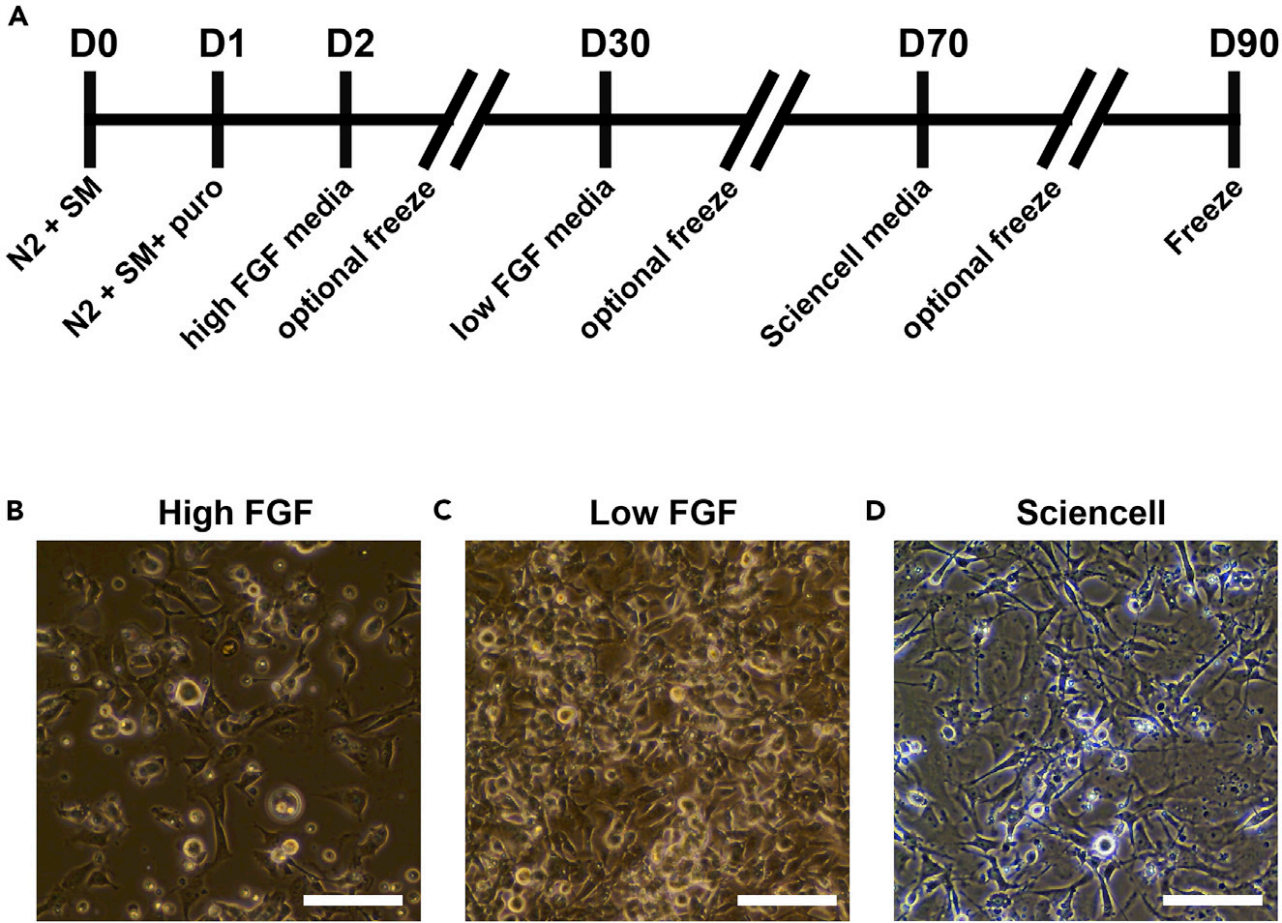
iAstrocyte Timeline and Representative Images (A) Timeline of iAstrocyte differentiation. (B) Representative image of iAstrocytes in high FGF media in mono-culture. Scale bar, 50 μm. (C) Representative image of iAstrocytes in low FGF media in mono-culture. Scale bar, 50 μm. (D) Representative mage of iAstrocytes in ScienCell astrocyte media. Scale bar, 50 μm.

***Note:*** The FBS, EGF, and FGF will push the NPC-like cells toward an astrocyte fate.

1. Thaw a vial of the previously NGN2-rTTA transduced hiPSCs and plate onto 3 wells of a 1:50 (Matrigel: DMEM:F-12) Matrigel-coated 6-well plate (7 × 10^4^ cells/cm^2^) in Stem MACS iPS-Brew XF media with 10 μM Y27632.
2. Proceed through the iNeuron differentiation as described above up through day 1 (Figures 1A and 2A). (Day 2) After 24 h, re-seed the cells (which are now at an NPC-like stage) at 1 million cells/well on a 1:20 Matrigel-coated 6-well plate (1 × 10^5^ cells/cm^2^) in high FGF Astrocyte media for first stage maintenance (Figure 2A).

3. Let grow with half media exchanges every 3 days and re-plate via the same method as for hiPSCs 1:2 when it reaches ∼90% confluency to 1:35 Matrigel-coated plates.

**CRITICAL:** High density is extremely important throughout differentiation. If the cells are too sparse, they will stop growing completely. This is especially true in the first two stages (high FGF media and low FGF media, up through day 70 of differentiation).

**Pause Point:** You can freeze down at this stage in high FGF astrocyte media + 10% DMSO.

***Note:*** Increased Matrigel dilution of 1:35 increases NPC adherence.

***Note:*** Cells will have a pyramidal shape with sparse, short processes (Figure 2B).

4. (Day 30) Exchange half media to low FGF Astrocyte media for second stage maintenance.
5. Continue to feed, let grow, and expand as before now in low FGF Astrocyte media for second stage maintenance up to day 69.

**Pause Point:** You can freeze down at this stage in low FGF astrocyte media + 10% DMSO.

***Note:*** Cells will have similar pyramidal shape as in the high FGF stage. However, in more sparse cultures, the cells can flatten out (Figure 2C).

6. (Day 70) Exchange half media to ScienCell astrocyte media.

7. Continue to perform half media exchanges in ScienCell astrocyte media, let grow, and expand to day 90.
  a. At this point, when you re-plate, you do not need to coat with Matrigel.
    **Pause Point:** You can freeze down at this stage in ScienCell astrocyte media + 10% DMSO at 1 million cells per vial.
  b. Cells are ready after day 90 for tri-culture.

***Note:*** Cells will have abundant processes and take on a more star-shaped astrocyte phenotype (Figure 2D).

8. Immunostain cells for nestin, glutamine synthetase, SOX9, and thrombospondin-1.
  a. Greater than 80% of the cells should be positive for nestin, glutamine synthetase and SOX9 and at least 50% of the cells should express thrombospondin-1.
  b. iAstrocytes can also be tested for calcium wave propagation and glutamate uptake.

***Note:*** glutamate uptake can be observed at this stage, but the iAstrocytes are still immature.

### Differentiate hiPSCs to Common Myeloid Progenitors

**Timing: 11–14 days**

This step will produce a stock of CD41+CD235+ common myeloid progenitors (CMPs) that are ready to be differentiated into iMicroglia. This section of the protocol was based on previously published work (Paluru et al., 2014).

9. Thaw a vial of hiPSCs and plate onto 3 wells of a 1:50 (Matrigel: DMEM:F-12) Matrigel-coated 6-well plate (7 × 10^4^ cells/cm^2^) in 2 mL/well Stem MACS iPS-Brew XF media with 10 μM Y27632.
10. Allow to grow until ∼80% confluency.

11. Once ∼80% confluent, split cells 1:6 to a 1:3 (Matrigel : DMEM/F12) Matrigel-coated 6-well plate.
  ***Note:*** The high concentration of Matrigel is necessary for the health of the mesoendothelial cells that are produced by the differentiation, which produce the non-adherent common myeloid progenitors.
  a. Remove Stem MACS and wash 1× with 20°C PBS.
  b. Aspirate media from stem cells and apply warm dispase (incubated in 37°C water bath for 15 min) at half the normal amount of media in the plate (i.e., 1 mL per well of a 6 well plate)
    ***Note:*** Dispase is used in this passaging stage to keep the cells in clumps. The Human Pluripotent Stem Cell Core at CHOP, who developed this method, found this to be effective for performing the differentiation.
  c. Incubate in 37°C 5% CO_2_ incubator until cells begin lifting from plate (roughly 5 min).
  d. Apply a volume of warm DMEM:F-12 equal to that of the dispase used.
  e. Wash cell clumps from bottom of plate by pipetting up and down slowly with a 5 mL serological pipette 2–3 times, making sure not to break the clumps.
  f. Place cells in an appropriately sized conical tube and spin for 5 min at 162 × *g*, 20°C.
  g. Resuspend in Stem MACS by slowly adding media to the tube and then gently pipetting up and down with a 10 mL pipette until the pellet has uniformly gone into suspension. Take a p1000 and slowly break up the colonies 3–5 times so that they are a uniform suspension of small clumps.
  h. Re-plate with 10 μM Y27632 at 1:15 ratio.

12. Feed daily (2 mL/well) until the cells reach ∼60% confluency. See timeline (Figure 3).

**Figure 3:**
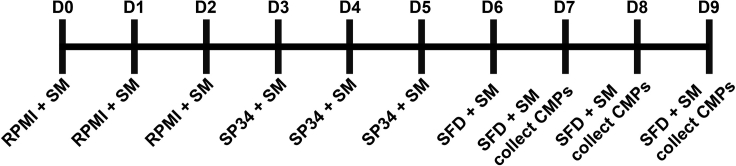
Timeline of CMP Differentiation
13. (Days 0–1) Once they reach ∼60% confluency, full exchange change media to RPMI with 5 ng/mL BMP4, 50 ng/mL VEGF, and 1 μM CHiR
14. (Day 2) Full exchange media with RPMI + 5 ng/mL BMP4, 50 ng/mL VEGF, and 20 ng/mL bFGF
  a. Wash once first with RPMI with no small molecules to remove dead cells
15. (Day 3) Full exchange media with SP34 + 5 ng/mL BMP4, 50 ng/mL VEGF, and 20 ng/mL bFGF
  a. Wash once first with RPMI with no small molecules to remove dead cells
16. (Days 4–5) Full exchange media each day with SP34 + 15 ng/mL VEGF and 5 ng/mL bFGF
17. (Day 6) Full exchange media with SFD + 50 ng/mL VEGF, 100 ng/mL bFGF, 50 ng/mL SCF, and 25 ng/mL Flt3L (day 6)
  a. Starting day 6, media is increased to 4 mL/well.
18. (Days 7–9) Full exchange media with SFD + 50 ng/mL VEGF, 100 ng/mL bFGF, 50 ng/mL SCF, and 25 ng/mL Flt3L. Fresh media with all components (2 mL/well) were added each day.

**CRITICAL:** From days 7 to 9, single cells shed off the adherent layer into the medium and are collected. These are the common myeloid progenitors (CMPS). Collect supernatant by pulling all the media from the wells and placing into a 50 mL tube. Centrifuge at 162 × *g* for 5 min at 20°C. Aspirate the supernatant. The cell pellet is often not visible, so leave roughly 500 μL of the supernatant to avoid aspirating the CMPs. Resuspend in 2 mL of SFD with small molecules. Feed the wells with the fresh media while spinning, and then add back the resuspended used media equally across the wells.

***Note:*** There is line-to-line variability on the number of CMPs produced. An efficient differentiation will yield 2 × 10^5^ to 5 × 10^5^ per well.

***Note:*** Cultures are terminated after day 9 as non-CMPs begin to be produced and reduce the purity.

19. CMPs are frozen at 1–3 million cells per vial in 90% FBS and 10% DMSO.

**Pause Point:** Frozen CMPs can be stored in LN2 until ready to start tri-culture.

**CRITICAL:** All three base media (RPMI, SP34, and SFD) are supplemented with 2 mM glutamine, 50 μg/mL ascorbic acid, and 400 μM monothioglycerol (MTG) fresh daily.

### Assemble the Tri-culture

**Timing: 30 days**

**Figure 4:**
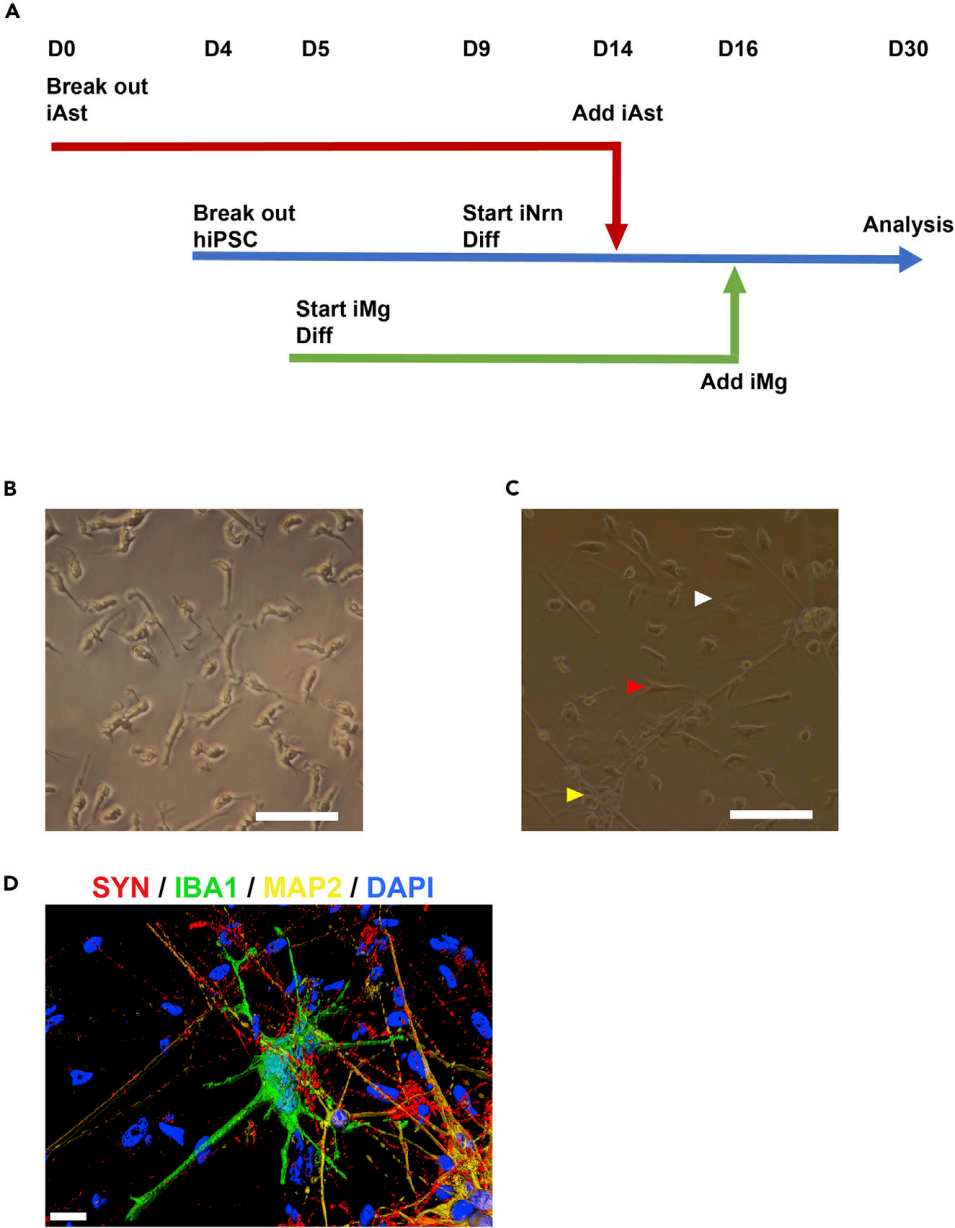
Timeline of Tri-culture Assembly and Representative Images of iMicroglia and Tri-culture (A) Timeline for assembling the 30-day tri-culture. (B) Representative image of D11 90 iMicroglia in mono-culture. Scale bar, 50 μm. (C) Bright-field image of tri-culture at D30. White arrowhead identifying an iAstrocyte. Red arrowhead identifying an iMicroglia. Yellow arrowhead identifying an iNeuron. Scale bar, 50 μm. (D) 3D reconstruction of IBA1+ (green) in contact with MAP2+ (yellow) dendrites that have SYN+ (red) synapses in a day 30 tri-culture. Scale bar, 20 μm.

This is a 30-day protocol that combines three differentiations. It is expected that the iPSC line for the iNeurons has already been transduced with the NGN2 virus, have had stocks frozen down, and have been tested for differentiation yield. It is also expected that the iAstrocyte differentiation is completed to day 90 and are frozen down. Lastly, it is expected that CMPs have been generated before the 30-day protocol begins. This protocol is designed to end on day 21 of the iNeuron differentiation, with the iAstrocytes added on day 5 and iMicroglia added on day 7 of the iNeuron differentiation (Figure 4A). This can be amended for extended cultures if desired. The iMicroglia differentiation in this section was based on previously published work (Abud et al., 2017). Time commitments per step are dependent on scale, number of lines, and number of conditions in the experiment. It is highly recommended you write out the timepoints of the protocol on a calendar before you begin.

20. (Day 0) Thaw and plate iAstrocytes on uncoated 6-well plate in ScienCell media (1 million cells/well).
  a. The iAstrocytes need roughly 2 weeks (line-to-line variability) to expand.
  b. Feed and expand in ScienCell media up to day 14 of the differentiation. Initial amount that is thawed is experiment dependent based on how many tri-culture wells are needed.
21. (Day 4) Thaw a vial of 2 × 10^6^ transduced hiPSCs in preparation for the iNeuron differentiation onto 3 wells of a 1:50 Matrigel-coated 6 well plate.
  a. Expand the hiPSCs as previously described to 2 1:50 Matrigel-coated 10-cm dishes.
  b. The goal is to have the hiPSCs at ∼60% confluency on 2 10-cm dishes at day 9 when the iNeuron differentiation will begin.

**CRITICAL:** This generally is enough time to have a single vial plated onto 3 wells of a 6 well plate, then expanded to 2 10-cm dishes reach ∼60% confluency. However, this needs to be empirically tested, as described above, as each line will have different growth rates and different transduction efficiencies. In addition, this will need to be scaled based on the needs of the experiment.

22. (Day 5) Begin iMicroglia differentiation by thawing CMPs and plating them at 3.3 × 10^5^ cells/well of a 24-well CellBIND plate (1.74 × 10^5^/cm^2^) in iMg media (RPMI + 10% FBS, 100 ng/mL IL-34, 25 ng/mL M-CSF, 50 ng/mL TGF-β1, pen/strep) for differentiation and maintenance.
  **CRITICAL:** Do not use a larger sized well than a 24-well even at the same seeding density. iMicroglia yield will exponentially drop. Smaller wells do work.
  ***Note:*** iMicroglia can also be plated onto 24 wells plates/glass or smaller with 1:35 Matrigel coating, but it will have a slightly lower yield than the CellBIND counterpart.
  a. Thaw CMPs in the same manner as hiPSCs.
  b. Thaw in a 37°C water bath until there is a pea-sized amount of ice left.
  c. Slowly add thawed CMP solution to 9 mL of warmed RPMI.
  d. Spin at 162 × *g* for 5 min at 20°C.
  e. Aspirate supernatant and resuspend pellet gently with a 5 mL pipette in iMg media (3.3 × 10^5^ cells/mL). Slowly pipette up and down until pellet has fully gone into suspension.
  f. Plate at 1 mL/well on CellBIND 24-well plate.
  g. Let cells rest for 48 h (days 0 and 1).
  h. Half media exchanges will be performed on days 2 and 5, and a full exchange on day 8 of iMicroglia differentiation.

23. (Day 7) Perform a half media exchange on iMicroglia.

**CRITICAL:** Cytokines in the iMg media (IL-34, M-CSF, and TGF-β1) are unstable, so they must be added fresh for each media exchange (day 2 of iMicroglia differentiation).

24. (Day 9) Begin iNeuron Differentiation.
  a. Exchange Stem MACS iPS-Brew with N2 media + small molecules (day 0 of iNeuron differentiation).
  b. hiPSCs should be ∼60% confluent.
25. (Day 10) Feed iMicroglia and continue iNeuron differentiation.
  a. Perform a half media exchange on iMg (day 5 of iMicroglia differentiation).
  b. Exchange N2 media + small molecules with fresh N2 media + small molecules supplemented with 5 μg/mL puromycin (day 1 of iNeuron differentiation).
26. (Day 11) Re-seed the iNeurons at 7.5 × 10^4^ cells/well of a 1:20 Matrigel-coated Nunc 8 Well chamber slide (∼1.07 × 10^5^ cells/cm^2^) in iN media for maintenance (day 2 of iNeuron differentiation).

***Note:*** Microglia will be highly ramified by the end of the 11-day differentiation (Figure 4B).

***Note:*** 8-well chamber slides will have 500 μL of media.

***Note:*** Tri-culture will be combined onto the iNeurons in the chamber slide. Tri-cultures can be combined onto different plate formats, but they have not been rigorously tested by this group.

27. (Day 13) Feed iMicroglia and add Ara-c to iNeurons.
  a. Perform a full exchange on the iMicroglia (day 8 of iMicroglia differentiation).
  b. Exchange 50% of iNrn media with iN media for maintenance supplemented with 4 μM Ara-c for a final concentration of 2 μM Ara-c (day 4 of iNeuron differentiation).

28. (Day 14) Remove Ara-c and introduce iAstrocytes (day 5 of iNeuron differentiation).
  a. First perform a full media exchange with iNeurons with iN media for maintenance to remove Ara-c.
    **CRITICAL:** The iNeurons are now very sensitive and can lift easily from the plate, so all media exchanges should be done very carefully.
  b. Re-seed iAstrocytes onto iNeurons at 5 × 10^4^ cells/well of an 8 well chamber slide ( ∼7.1 × 10^4^ cells/cm^2^)

**CRITICAL:** Resuspend iAstrocytes in ScienCell astrocyte media to 5 × 10^4^ cells/100 μL. Survivability in the tri-culture increases if they are re-seeded in a mixed media initially.

***Note:*** The iAstrocytes are added as early as possible to help support and mature the iNeurons, but it must be added after Ara-c treatment is removed, otherwise they will die.

29. (Day 16) Re-seed iMicroglia at 1 × 10^5^/well of the 8 well chamber slide (∼1.43 × 10^5^ cells/cm^2^) (day 11 of iMicroglia differentiation) (day 7 of iNeuron differentiation).
  a. Lift iMicroglia into a single cell suspension with Accutase.

**CRITICAL:** iMicroglia yield (total number of cells after lifting) can dramatically decrease with larger tubes. Use as small a tube (preferentially 2 mL Eppendorf tubes) as possible to increase yield.

**CRITICAL:** Resuspend iMicroglia in iMg media to 1 × 10^5^

cells/100 μL. Survivability in the tri-culture increases if they are re-seeded in a mixed media initially.

***Note:*** Yield for iMicroglia from CMPs is usually around 50%–60% of total number of CMPs seeded but varies across lines.

***Note:*** iMicroglia can be differentiated beforehand and frozen. However, the yield will be reduced due to cell death during freeze/thaw process.

30. (Day 21) Perform a half media exchange with iN media without doxycycline (day 12 of iNeuron differentiation).
31. (Day 26) Perform a half media exchange with iN media without doxycycline (day 17 of iNeuron differentiation).
32. (Day 30) End of differentiation (day 21 of iNeuron differentiation) (Figures 4C and 4D).

***Note:*** Tri-culture can be extended beyond this timepoint. This group has extended iNrn differentiations to upwards of day 80. However, these were grown on rat astrocytes and were switched over to BrainPhys media (STEMCELL Technologies #05793) starting day 21 of iNeuron differentiation.

## Expected Outcomes

The iNeurons should be at >90% pure with expression of MAP2, synaptophysin, and PSD-95 (Ryan et al., 2020; Zhang et al., 2013). The iAstrocytes have higher variability in purity but >80% should express SOX9, glutamine synthetase, and nestin. Roughly 50% should express thrombospondin-1 by immunostaining (Ryan et al., 2020). Thrombospondin-1 does not come on until late stage differentiation in ScienCell media. The CMPs should be CD41+ CD235+. The iMicroglia should be >90% pure by expression of CX3CR1, TMEM119, IBA1, and P2RY12 by immunostaining (Ryan et al., 2020).

Roughly 1 million CMPs, astrocytes, and neurons will produce 5 to 6 tri-cultures in the 8-well chamber slide system due to the 50%–60% yield of microglia from CMPs. By scRNA-seq data (Ryan et al., 2020), with the described seeding densities, the tri-cultures will have a cell ratio of roughly 3.7:1:1 neurons to astrocytes to microglia. These tri-cultures are quiescent and can produce an inflammatory response if exposed to a stimulus such as viral infection. In addition, the cells in the culture exhibit physiological functions including synaptic phagocytosis and cytokine production by the iMicroglia, synapses and action potentials by the iNeurons, and *GLT-1* expression and neuronal support by the iAstrocytes (Ryan et al., 2020). Tri-cultures can be extended beyond the 30 days described in this protocol.

## Limitations

Due to the relatively low yields of CMPs and inability to expand the iMicroglia, scaling the tri-culture up may be difficult or at the very least require large investments in time and reagents. Depending on the line, some hiPSC lines can produce less homogenous populations of CD41+CD235+ CMPs. One may need to FACS sort to ensure the non-adherent cells are CMPs. In addition, changes in confluency or Matrigel concentration can reduce yields of CMPs. Tri-cultures are extremely delicate and excessive shaking, moving, or too forceful media exchanges can cause the neurons to lift from the plate.

## Troubleshooting

### Problem

Transduced iPSCs have not grown to proper confluence by day 4 of the tri-culture differentiation.

## Potential Solution

If the timing is off for growing the NGN2 rTTA transduced cells, it is acceptable to allow the iMicroglia or iAstrocytes to stay in mono-culture, even past day 11 for the iMicroglia. iMicroglia were maintained in mono-culture up to 15 days past D11 with no signs of increased death or cell stress. However, we have not tested the efficacy of the tri-culture if the iAstrocytes or iMicroglia are added later than described. There may be a decrease in maturity of the iNeurons without the iAstrocytes.

## Problem

Astrocyte differentiation fails.

## Potential Solution

The iAstrocyte differentiation is very sensitive to density. Too low of a density and the iAstrocytes will flatten out and stop dividing. It is imperative to not split more than 1:2 throughout the differentiation. After day 90, they can be split upwards of 1:5, but this can vary from line to line.

## Problem

iMicroglia are lost during collection.

## Potential Solution

When the iMicroglia are lifted from their CellBIND plates, it is imperative to use the smallest tube possible to centrifuge the cells. This may require using less PBS or wash media to quench the Accutase. It is also necessary to use a bucket rotor and not one at a fixed angle, as in the fixed angle centrifuges, the cells will not properly pellet at the bottom of the tube, leading to loss of iMicroglia. CMPs plated to 24 well or smaller work very effectively. Larger plate sizes (i.e., 12 well and 6 well) have a massive drop in iMicroglia yield even accounting for seeding density for the larger surface area. This creates an issue when lifting the cells for the tri-culture as it requires more wells, more Accutase, and more time.

## Problem

Low transduction rates for hiPSC lines with NGN2 and rTTA lentiviruses

## Potential Solution

Some lines have low transduction rates for the NGN2 and rTTA lentiviruses. We have found that using Lentiblast (Oz Biosciences LB00500) has increased efficiency.

## Problem

Low yield of iNeurons, iAstrocytes, or iMicroglia post re-plating.

## Potential Solution

There will be some line variability for survival during re-plating for each of the cell types. This was most apparent with iNeuron and iAstrocyte differentiations. The growth and survival rates need to be determined empirically. However, the described seeding densities and timelines have worked for at least three lines for each of the three differentiations.

## Problem

CMPs do not attach to plate after 1 day.

## Potential Solution

The CMPs attach poorly in wells with large surface areas (larger than a 24 well). Collect the media from the well and re-plate onto a smaller, Matrigel-coated plate or cellBIND plate. Let rest for 48 h and then proceed from the day 2 step with a half media exchange.

## Problem

iNeurons lift during differentiation, fixation, or immunostaining:

## Potential Solution

The iNeurons become more fragile and lift more easily form the plate as they differentiate and grow. If the iNeurons lift, unfortunately they are not salvageable. Be gentle when exchanging media, travel as small a distance with the plates as possible, and do not fully remove media when fixing and performing immunostaining. To compensate for the remaining liquid during washes during fixation or immunostaining, we recommend at least 10-min washes.

## Problem

Phenotypes are not present in disease models using this tri-culture.

## Potential Solution

The iAstrocytes and iNeurons have relatively immature phenotypes (Ryan et al., 2020). Extending the cultures or utilizing other differentiations for the individual cell types may result in a more mature phenotype (Nehme et al., 2018; Tchieu et al., 2019; Wang et al., 2013) and thus a more physiologically relevant system depending on what question is being asked.

## Resource Availability

### Lead Contact

Further information and requests for resources and reagents should be directed to and will be fulfilled by the Lead Contact, Stewart Anderson (sande@pennmedicine.upenn.edu).

### Materials Availability

This study did not generate new, unique reagents.

### Data and Code Availability

The datasets generated during and/or analyzed during this study are available in the GEO repository https://www.ncbi.nlm.nih.gov/geo/query/acc.cgi?acc=GSE143687. The accession number for the data reported in this paper is GEO:GSE143687.
